# Sleep disturbance and intrusive memories after presenting to the emergency department following a traumatic motor vehicle accident: an exploratory analysis

**DOI:** 10.1080/20008198.2018.1556550

**Published:** 2019-01-14

**Authors:** Annemarie I. Luik, Lalitha Iyadurai, Isabel Gebhardt, Emily A. Holmes

**Affiliations:** a Sleep and Circadian Neuroscience Institute, Nuffield Department of Clinical Neurosciences, University of Oxford, Oxford, UK; b Department of Epidemiology, Erasmus MC University Medical Centre, Rotterdam, Netherlands; c Department of Psychiatry, University of Oxford, Oxford, UK; d Institute of Psychology, Heidelberg University, Heidelberg, Germany; e Division of Psychology, Department of Clinical Neuroscience, Karolinska Institutet, Stockholm, Sweden

**Keywords:** Intrusive memories, trauma, posttraumatic stress disorder, sleep, early intervention, recuerdos intrusivos, trauma, trastorno de estrés postraumático, sueño, intervención temprana, 闯入记忆，创伤，创伤后应激障碍，睡眠，早期干预

## Abstract

**Background**: Sleep disturbances are common after traumatic events and have been hypothesized to be a risk factor in the development of psychopathology such as that associated with posttraumatic stress disorder (PTSD).

**Objective**: To assess the association between intrusive memories, a core clinical feature of PTSD, and self-reported sleep disturbance shortly after experiencing or witnessing a motor vehicle accident, and whether a brief behavioural intervention (trauma reminder cue and Tetris gameplay) reduced sleep disturbance post-trauma.

**Method**: The exploratory analyses included 71 participants (mean age 39.66, standard deviation 16.32; 37 women, 52.1%) enrolled in a previously published proof-of-concept randomized controlled trial. Participants were recruited from the emergency department after experiencing or witnessing a traumatic motor vehicle accident. Intrusive memories were assessed with a daily paper-and-pen diary for one week post-trauma, and sleep disturbances with three questions from the Impact of Event Scale-Revised assessing problems initiating sleep, problems maintaining sleep and dreams about the event at one week and one month post-trauma. Missing data were imputed 15 times.

**Results**: The total number of intrusive memories during the first week post-trauma suggested weak to moderate pooled intercorrelations with problems initiating and maintaining sleep. An ordinal regression using imputed data suggested that the intervention had no effect on sleep disturbances, while completers only analyses suggested an improvement in problems maintaining sleep at one week.

**Conclusions**: This exploratory study suggested that experiencing early intrusive memories is related to sleep disturbances. Sleep disturbance might be a particularly important construct to assess in studies involving intrusive memories post-trauma.

## HIGHLIGHTS

• Sleep disturbances are common after trauma.• We assessed the association between intrusive memories and sleep, and whether a brief behavioural intervention targeting intrusive memories (trauma reminder cue and Tetris gameplay) affected sleep post-trauma.• More intrusive memories post-trauma are potentially related to a greater severity of sleep disturbances.• The intervention had mixed effects on the experience of sleep disturbances.• Sleep disturbance might be a particularly important construct to assess in studies involving intrusive memories post-trauma.

## Abstract (es)

**Antecedentes**: Las alteraciones del sueño son frecuentes tras eventos traumáticos y han sido planteados como un factor de riesgo para el desarrollo de psicopatología como la que está asociada con el trastorno de estrés postraumático (TEPT).

**Objetivo**: Evaluar la asociación entre recuerdos intrusivos, característica clínica central del TEPT, y alteraciones del sueño auto-reportadas, al poco tiempo luego de experimentar o ser testigo de un accidente automovilístico, y si una intervención conductual breve (estímulos gatillantes de recuerdos traumáticos, y el juego Tetris) reduce alteraciones del sueño postrauma.

**Método**: Los análisis exploratorios incluyeron 71 participantes (edad promedio 39.66, desviación estándar 16.32; 37 mujeres, 42.1%) incluidos en ensayo clínico randomizado previamente publicado. Los participantes fueron reclutados desde el departamento de emergencias tras experimentar o haber sido testigo de un accidente automovilístico traumático. Los recuerdos intrusivos fueron evaluados diariamente con papel y lápiz a través de un diario durante 1 semana postrauma, y las alteraciones del sueño con 3 preguntas provenientes de la Escala Revisada de Impacto del Evento evaluando problemas para conciliar el sueño, problemas para mantener el sueño, y sueños sobre el evento a la primera semana y al primer mes postrauma. Datos faltantes fueron imputados 15 veces.

**Resultados**: El número total de recuerdos intrusivos durante la primera semana postrauma sugirieron inter-correlaciones leves a moderadas con alteraciones de conciliación y mantención del sueño. Una regresión ordinal que utilizó datos imputados sugirió que la intervención no tuvo efecto en las alteraciones del sueño, mientras que los análisis ‘completers-only’ sugirieron una mejoría en las alteraciones de mantención del sueño a la semana 1.

**Conclusiones**: Este estudio exploratorio sugirió que la experimentación de recuerdos intrusivos está relacionada con alteraciones del sueño. Las alteraciones del sueño pueden ser un constructo particularmente importante, a evaluar en estudios que involucran recuerdos intrusivos postrauma.

## Abstract (zh)

**背景：**睡眠障碍在创伤事件后很常见，并且被假设为精神病理学发展的风险因素，例如与创伤后应激障碍（PTSD）相关的疾病。

**目的：**评估闯入性记忆（创伤后应激障碍的核心临床特征），以及在经历或目睹机动车事故后不久自我报告的睡眠障碍之间关联，以及短暂的行为干预（创伤提醒线索和俄罗斯方块游戏）是否减少创伤的睡眠障碍。

**方法：**探索性分析包括71名被试（平均年龄39.66岁，标准差16.32; 37名女性，52.1％）参加先前已发表的概念验证（proof-of-concept ）随机对照试验。从急诊室招募了经历或目睹创伤性机动车事故后的被试，创伤后1周内的用纸笔日记评估闯入性记忆。

在创伤后一周和一个月之后通过《事件影响量表修订版》的3个问题评估睡眠障碍 （ 进入睡眠的问题;维持睡眠的问题，和梦到相关事件）。对缺失数据进行了15次插补。

**结果：**创伤后第一周内创入性记忆的总数和，在进入睡眠和维持睡眠的问题存在轻度到中度的综合相关系（pooled intercorrelations）。插补数据进行序数回归表明干预对睡眠障碍没有影响，而完成者分析（completers only analyses）表明在1周时维持睡眠的问题有所改善。

**结论：**这项探索性研究表明，体验早期闯入性记忆与睡眠障碍有关。在涉及创伤后侵入性记忆的研究中，可以将睡眠障碍作为一个特别重要的结构进行评估。

**Abbreviations**: PTSD: Post-Traumatic Stress Disorder; M: Mean; SD: Standard Deviation.

Sleep disturbances are commonly experienced after traumatic events (Lavie, 2001). Recently it has been hypothesized that sleep is also an important component in the development of posttraumatic stress disorder (PTSD) (Sinha, 2016), as well as a risk factor for other mental health disorders (Pigeon, Bishop, & Krueger, 2017). Indeed, a study following 102 people who experienced a motor vehicle accident demonstrated that PTSD after one year was predicted by sleep disturbances at one month (Koren, Arnon, Lavie, & Klein, 2002).

A core clinical feature of acute stress disorder and PTSD is experiencing recurrent, intrusive and involuntary distressing memories of the traumatic event (American Psychiatric Association, 2013). The occurrence of intrusive memories early after a traumatic event has been associated with symptoms of PTSD (Bryant et al., 2017) and might be a modifiable target for early intervention and prevention (Holmes, James, Coode-Bate, & Deeprose, 2009; Iyadurai et al., 2018).

Key to the current paper, it has also been suggested that the experience of intrusive memories of trauma is linked to sleep by studies using experimental sleep deprivation (Kleim, Wysokowsky, Schimd, Seifritz, & Rasch, 2016; Porcheret, Holmes, Goodwin, Foster, & Wulff, 2015). Moreover, recent work also demonstrates an association between intrusive memories and nightmares (Birkeland & Heir, 2017). Mechanistically-driven psychological interventions (Holmes et al., 2018) delivered soon after a traumatic event could therefore potentially provide a valuable opportunity to not only reduce the number of intrusions experienced (Iyadurai et al., 2018), but also to reduce sleep disturbance and development of mental health disorders in the future. However, as yet, there has been no examination of the relationship between early intrusive memories and sleep disturbance after a real life traumatic event.

Based on the proposition that visuospatial cognitive tasks act not merely via distraction on intrusive memories but by modality-specific interference with sensory (visual) aspects of intrusive memory, a brief behavioural intervention involving a reminder cue and Tetris gameplay shortly after the traumatic event has been tested to reduce the number of intrusive memories (Holmes et al., 2009; Horsch et al., 2017, Iyadurai et al., 2018). Using a publicly available dataset from a proof-of-concept randomized controlled trial which found that this behavioural intervention reduced intrusive memories over the first week post-trauma (Iyadurai et al., 2018), we aimed to (1) describe the association between the number of intrusive memories over the first week post-trauma and self-reported trouble initiating sleep, trouble maintaining sleep and dreams about the traumatic event at both one week and one month post-trauma, (2) assess whether a simple one session behavioural intervention (reminder cue and Tetris gameplay) reduces sleep disturbance at one week and one month and (3) examine whether a potential reduction of sleep disturbance is dependent on the number of intrusive memories experienced in the first week.

## 1. Method

### 1.1. Participants

The current study analyses data from 71 participants (mean [*M*] age 39.66, standard deviation [*SD*] 16.32; 37 women, 52.1%; 56 white British, 78.9%) recruited from an emergency department after experiencing or witnessing a traumatic motor vehicle accident (Iyadurai et al., 2018). Data are publicly available via the Open Science Framework (https://osf.io/e4hc7/). Inclusion criteria (e.g. experienced or witnessed a motor vehicle accident, met the DSM-IV-TR criterion A1 for a traumatic event) and exclusion criteria (e.g. history of severe mental illness, current intoxication or neurological condition) for these analyses can be found in Supplemental Table 1. Written informed consent was obtained before completing the baseline assessment. The study received ethical approval from the local National Research Ethics Service Research Ethics Committee (Oxford C: 12/SC/0485).

**Table 1.** Descriptive information about the sample (*N* = 71) for the intervention and control groups.

		Intervention (*n *= 37)				Control (*n *= 34)			
		*M*	*SD*	*n*	%	*M*	*SD*	*n*	%
*Baseline*									
Age (years)		38.9	16.1			40.5	16.8		
Women				20	54.1%			17	50.0%
Ethnicity	White British			28	75.7%			28	82.4%
	Ethnic minority			9	24.3%			6	17.6%
Perceived life threat to self		5.19	3.20			5.56	3.23		
*Week 1 after index traumatic event*									
Total intrusive memories		8.73	11.55			10.70	7.29		
Problems initiating sleep	Not at all			20	54.1%			14	41.2%
	A little bit			6	16.2%			4	11.8%
	Moderately			3	8.1%			5	14.7%
	Quite a bit			5	13.5%			7	20.6%
	Extremely			0	0.0%			3	8.8%
Problems maintaining sleep	Not at all			19	51.4%			12	35.3%
	A little bit			7	18.9%			7	20.6%
	Moderately			5	13.5%			2	5.9%
	Quite a bit			3	8.1%			10	29.4%
	Extremely			0	0.0%			2	5.9%
Dreams about event	Not at all			23	62.2%			22	64.7%
	A little bit			8	21.6%			2	5.9%
	Moderately			3	8.1%			3	8.8%
	Quite a bit			0	0.0%			4	11.8%
	Extremely			0	0.0%			2	5.9%
*Month 1 after traumatic event*									
Problems initiating sleep	Not at all			19	51.4%			17	50.0%
	A little bit			7	18.9%			5	14.7%
	Moderately			2	5.4%			7	20.6%
	Quite a bit			1	2.7%			1	2.9%
	Extremely			1	2.7%			1	2.9%
Problems maintaining sleep	Not at all			19	51.4%			16	47.1%
	A little bit			7	18.9%			7	20.6%
	Moderately			3	8.1%			3	8.8%
	Quite a bit			1	2.7%			3	8.8%
	Extremely			1	2.7%			2	5.9%
Dreams about event	Not at all			25	67.6%			22	64.7%
	A little bit			5	13.5%			6	17.6%
	Moderately			0	0.0%			1	2.9%
	Quite a bit			0	0.0%			1	2.9%
	Extremely			1	2.7%			1	2.9%

Data before imputation are presented. *M* = Mean; *SD* = Standard deviation.

### 1.2. Material and procedure

Between March 2014 and January 2015, potential participants were identified by emergency department staff. Eligibility was assessed and a description of the study was provided to prospective participants. A 1:1 randomization stratified for gender, age and perceived life-threat to self was used to assign participants to the intervention or control condition. Time between trauma and consent was 192 minutes (*SD* = 69) in the intervention group and 211 minutes (*SD* = 67) in the control group. After one week and one month, participants were assessed for follow-up.

#### 1.2.1 Intervention

In the intervention condition, participants received memory reminder instructions (think back to the event and briefly tell the researcher the worst moments) before practice in playing the computer game Tetris (emphasizing mental rotation). They then played for about 20 minutes in total with at least one uninterrupted period of 10 minutes. In the control condition, participants completed an activity log about their time spent in the emergency department for a similar duration. Condition-specific procedures above were delivered around usual care at the emergency department. More details can be found in Iyadurai et al. (2018).

#### 1.2.2 Measures

Various measurements were completed by the participants in the original study (Iyadurai et al., 2018). Here we will only describe measures used for the analyses reported in this manuscript.

##### Intrusive memories of the traumatic event

Intrusive memories of the traumatic event were measured for one week with a daily paper-and-pen diary (e.g. Holmes et al., 2009). Intrusive memories were described as ‘image-based memories of the accident that pop into your mind without warning’. The diary comprised simple tick boxes for the day and time period (morning/afternoon/evening), and daily SMS reminders were sent (see Iyadurai et al., 2018). Occurrence of intrusive memories was reported from the day of the traumatic event for one week and summed to give a total score for the whole week.

#### Sleep disturbance

Sleep disturbance was measured with three single items that are part of the Impact of Event Scale-Revised (Creamer, Bell, & Failla, 2003). Problems initiating sleep were measured by rating the statement ‘I had trouble falling asleep’ (item 15), problems maintaining sleep by rating ‘I had trouble staying asleep’ (item 2) and dreams of the traumatic event by rating ‘I had dreams about it’ (item 20). All statements were rated as on a 5-point scale anchored with *Not at all*, *A little bit*, *Moderately*, *Quite a bit* or *Extremely* scored as 0 to 4, respectively.

#### Perceived life threat during the traumatic event

Threat to self during the traumatic event was measured with the question ‘To what extent did you feel your life was in danger?’ rated on a 0 (*not at all*) to 10 (*extremely*) scale (Blanchard, Hickling, Taylor, & Loos, 1995).

#### Demographics

Age and gender were obtained during the baseline examination.

### 1.3. Data analyses

All analyses reported were exploratory in nature and not formulated at the time the study was designed. Data were missing for four participants (5.6%) at one week and for nine participants (12.7%) at one month. Missing data were imputed by creating 15 datasets, using age, sex and threat to self as auxiliary variables, in line with the original manuscript (Iyadurai et al., 2018). To assess whether intrusive memories and sleep disturbances were related, Spearman’s rank correlations were calculated to account for the ordinal nature of the sleep variables across groups and also for the control group separately to examine the relationship independent of a possible intervention effect. An ordinal regression was used to assess the effect of the intervention on sleep disturbance. To assess whether the number of intrusive memories during one week after the trauma accounted for any potential effect of the intervention on sleep disturbance we controlled for intrusive memories in a second model. As sensitivity analyses, all analyses were repeated using only completers data. Analyses were performed using IBM SPSS version 24.0 (IBM Corp., Somers, NY, USA).

## 2. Results

A total of 71 participants was randomized to the intervention (*n* = 34) and to the control group (*n* = 34). Descriptive information can be found in Table 1.

Pooled Spearman’s rank intercorrelations suggested weak to moderate associations between the number of intrusive memories (total for first week) and problems initiating (*r *= .34, *p *< .01) and maintaining sleep (*r *= .37, *p *< .01), and dreams of the traumatic event (r = .26, *p* < .01) at one week and maintain sleep at one month (*r *= .30, *p *< .05), see Table 2. When analysing intercorrelations in the control group only, these correlations were lower (range *r *= .26 to .29). Using a completers only dataset, the intercorrelations also suggested weak to moderate associations between the number of intrusive memories and sleep disturbances at one week and one month (range *r *= .27 to .40, all *p*s < .05, see Supplemental Table 2).

**Table 2.** Pooled Spearman’s rank correlations of early number of intrusive memories (total of first seven days post-trauma) with sleep disturbances reported at one week and one month.

	1	2	3	4	5	6	7
1. Intrusive memories at one week	–	.34**	.37**	.26	.20	.30*	.20
2. Problems initiating sleep at one week		–	.78**	.49**	.43**	.33**	.42**
3. Problems maintaining sleep at one week			–	.57**	.47**	.50**	.53**
4. Dreams of traumatic event at one week				–	.39**	.39**	.59**
5. Problems initiating sleep at one month					–	.75**	.58**
6. Problems maintaining sleep at one month						–	.61**
7. Dreams of traumatic event at one month							–

*p < .05, **p < .01.

The intervention effect on sleep disturbance was assessed with an ordinal regression, which indicated that the intervention did not reduce sleep problems at one week and one month, see Table 3. When analysing only those who completed the surveys, the intervention reduced problems maintaining sleep at one week by one response category (B = −1.00, 95% Confidence Interval (CI) [−1.90, −0.09]) when compared with the control intervention (see Supplemental Table 3, model 1).

**Table 3.** Pooled estimates for the effects of one intervention session on day of the traumatic event on sleep disturbances at week 1 using ordinal regression.

	Problems initiating sleep		Problems maintaining sleep		Dreams of traumatic event	
	B	95% CI	B	95% CI	B	95% CI
*Model 1*						
Intervention^a^	−0.70	−1.62, 0.22	−0.84	−1.79, 0.11	−0.25	−1.25, 0.75
*Model 2*						
Intervention^a^	−0.48	−1.44, 0.49	−0.63	−1.61, 0.35;	−0.05	−1.13, 1.02
Total intrusions	0.02	0.00, 0.04	0.03	0.01, 0.05	0.01	0.00, 0.03

^a^Reference is control group.

To assess whether the intervention effect on problems maintaining sleep in the completers only analyses was explained by intrusive memories, the total number of intrusive memories was added in model 2. Adding intrusive memories caused a 23% reduction in effect size (B = −0.77, 95% CI [−1.72, 0.17]) for intervention, removing the significant effect on problems maintaining sleep (see Supplemental Table 3, model 2).

## 3. Discussion

These exploratory analyses, aimed at exploring the yet unexamined association between early intrusive memories and sleep disturbance after a traumatic event, suggested that intrusive memory frequencies over one week, directly after experiencing or witnessing a motor vehicle accident, are related to sleep disturbances, in particular initiating and maintaining sleep, at one week and one month. As results are mixed with regards to the effect of the intervention on sleep disturbances, we are unable to determine whether a one session simple behavioural intervention on the day of the traumatic road accident while waiting in the Emergency Department, including a reminder cue and Tetris gameplay, can help to reduce subsequent sleep disturbances and whether this would be dependent on the reduction of intrusive memories.

The association of intrusive memories with sleep disturbances could be explained in multiple ways and the association is potentially bidirectional. First, intrusive memories of a traumatic event (e.g. mental image of blood dripping) during the night could potentially keep patients awake. Circadian variation in related concepts such as mood has been demonstrated (McClung, 2013), a potential 24-hour rhythm of intrusive memories has not been studied formally to our knowledge. Second, the association could be explained by hyperarousal of the central nervous system, which might prevent patients from sleeping. In cognitive models, the experience of having an unwanted intrusive memory of a trauma leads to increased arousal such as an increased heart rate and sweating, which can lead the system to be hyperaroused (Ehlers & Clark, 2000). Third, one of the core diagnostic criteria of PTSD, and part of the same DSM-5 criteria cluster as intrusive memories, is experiencing nightmares (American Psychiatric Association, 2013). Recently, a high interconnectedness between nightmares and intrusive thoughts was indicated when assessing the interconnectedness between symptoms of PTSD (Birkeland & Heir, 2017). This is in line with our observation that experiencing more intrusions is potentially related to more dreams about the event, offering further potential to disturb sleep. However, the intervention did not appear to improve nightmares, potentially due to a low occurrence of nightmares in the current study. Fourth, PTSD has been hypothesized to be a memory disorder (Van Marle, 2015). Sleep disturbance is known to have effects on cognition (Van Someren et al., 2015), although the effect on intrusive memories, after for example sleep deprivation, are conflicting (Kleim et al., 2016; Porcheret et al., 2015).

Our results are mixed with regards to the effect of the intervention on sleep disturbances, potentially due to limited power. Attrition could have further led to an underestimation or overestimation of the results by those having a more severe response to the trauma dropping out. If a short intervention would be able to reduce problems with sleep, this might be important for preventative indications for the development of PTSD (Bryant et al., 2017; Ho, Chan, & Tang, 2016) and potentially other mental health disorders (Freeman et al., 2017; Pigeon et al., 2017). In the completer only analysis, a reduction in problems maintaining sleep in the intervention group was found. This reduction was dependent on the number of intrusive memories, suggesting that if any benefits of the intervention on sleep exist they are related to a reduction in intrusive memories. We want to emphasize that the results should be interpreted with caution – analyses using multiple imputations did not demonstrate an effect and the current study is exploratory, potentially not adequately powered to answer these post-hoc formulated research questions. Further research, specifically assessing sleep disturbances when treating intrusive memories, is needed to determine whether sleep disturbances can be prevented by treating intrusive memories.

The current study was an exploratory study which employed a publicly available dataset. Although using publicly available data is advantageous, it also carries several limitations. First, the original data was obtained during a proof-of-concept study with a relatively small sample size not powered for the current analysis. This led to small cell sizes, that is the frequency of participants who endorsed at least moderate sleep complaints is relatively low in the sample, which can lead to underestimation or overestimation of the associations and a potentially larger influence from the multiple imputations. In addition, data were collected before the current hypotheses were formulated and thus no formal validated measures of sleep disturbances were included in the study. Although we are aware that using ordinal, single item measures can create difficulties, it has recently been suggested that single item measures from large questionnaires can give a sufficient prediction of sleep disturbances (Hughes, Ulmer, Gierisch, Mid-Atlantic, & Howard, 2018). Only two people reported the use of anxiolytics, anti-depressants or sleep medication at one week, hence we were not able to examine the impact of medication on sleep disturbance. Lastly, the study did not involve any baseline sleep measurement before patients presented to hospital, making it impossible to assess whether the reported sleep disturbances were already present before the motor vehicle accident. Additionally, within the control group a substantial amount of participants at week 1 did not endorse any sleep problems, which might have led to an underestimation of the effect size.

In conclusion, this exploratory study suggests that early experience of intrusive memories is related to sleep disturbance at both one week and one month and that sleep disturbance is potentially reduced by a simple one session intervention targeting acute intrusive memories, after experiencing or witnessing a traumatic event and while waiting in the hospital Emergency Department. In addition, sleep disturbance might be a particularly important construct to assess in studies involving traumatic events as it has previously been demonstrated to be an important factor in the development of PTSD.

## Supplementary Material

Supplemental Material
