# Supplementary material for: Sleep disturbance and intrusive memories after presenting to the emergency department following a traumatic motor vehicle accident: an exploratory analysis
Source: Eur J Psychotraumatol. 2019 Jan 14;10(1):1556550. doi: 10.1080/20008198.2018.1556550 (PMC6338269; doi:10.1080/20008198.2018.1556550)
Supplement: Supplemental Material [file ZEPT_A_1556550_SM2078.zip › SleepTraumaSupplementalTable1.docx]

Supplemental Table 1. Inclusion and exclusion criteria.

| Inclusion criteria | - Age ≥18 years - Experienced/witnessed a motor vehicle accident (as a driver, passenger, motorcyclist or pedestrian) - Meeting Diagnostic and Statistical Manual of Mental Disorders 4th Edition (DSM-IV) PTSD criterion A1 for a traumatic event ('experienced, witnessed or was confronted with an event or events that involved actual or threatened death or serious injury') - Seen in emergency department within 6 h of leaving scene of the accident - Reported memory of the accident - Fluent in written and spoken English - Alert and orientated, Glasgow Coma Scale score=15; - Sufficient physical mobility to play a computer game on the intervention platform (Nintendo DS) at the point of taking informed consent |
| --- | --- |
| Exclusion criteria | - Loss of consciousness for >5 min, - Reported history of severe mental illness, - Current intoxication, substance abuse or neurological condition, or currently suicidal. |
