# Supplementary material for: Sleep disturbance and intrusive memories after presenting to the emergency department following a traumatic motor vehicle accident: an exploratory analysis
Source: Eur J Psychotraumatol. 2019 Jan 14;10(1):1556550. doi: 10.1080/20008198.2018.1556550 (PMC6338269; doi:10.1080/20008198.2018.1556550)
Supplement: Supplemental Material [file ZEPT_A_1556550_SM2078.zip › SleepTraumaSupplementalTable2.docx]

Supplemental Table 2. Spearman’s rank correlations of early number of intrusive memories (total of first 7 days post-trauma) with sleep disturbances reported at 1 week and 1 month for completers only.

|  | 1 | 2 | 3 | 4 | 5 | 6 | 7 |
| --- | --- | --- | --- | --- | --- | --- | --- |
| 1. Intrusive memories  at 1 week | - | .36** | .40** | .27* | .29* | .39** | .33** |
| 2. Problems initiating sleep  at 1 week |  | - | .83** | .48** | .51** | .37** | .47** |
| 3. Problems maintaining sleep  at 1 week |  |  | - | .58** | .54** | .55** | .60** |
| 4. Dreams of traumatic events  at 1 week |  |  |  | - | .39** | .38** | .61** |
| 5. Problems initiating sleep  at 1 month |  |  |  |  | - | .75** | .47** |
| 6. Problems maintaining sleep  at 1 month |  |  |  |  |  | - | .57** |
| 7. Dreams of traumatic events  at 1 month |  |  |  |  |  |  | - |

**p*<.05, ***p*<.01
