# Supplementary material for: Sleep disturbance and intrusive memories after presenting to the emergency department following a traumatic motor vehicle accident: an exploratory analysis
Source: Eur J Psychotraumatol. 2019 Jan 14;10(1):1556550. doi: 10.1080/20008198.2018.1556550 (PMC6338269; doi:10.1080/20008198.2018.1556550)
Supplement: Supplemental Material [file ZEPT_A_1556550_SM2078.zip › SleepTraumaSupplementalTable3.docx]

Supplemental Table 3. Ordinal regression assessing effects of one session intervention on day of the traumatic event while in the hospital Emergency Department on sleep disturbances at week 1 for completers only.

|  | Problems initiating sleep | | Problems maintaining sleep | | Dreams of traumatic event | |
| --- | --- | --- | --- | --- | --- | --- |
|  | B | 95% CI | B | 95% CI | B | 95% CI |
| *Model 1* |  |  |  |  |  |  |
| Intervention^1^ | -0.83 | -1.74, 0.08 | -1.00 | -1.90, -0.09 | -0.37 | -1.37, 0.63 |
| *Model 2* |  |  |  |  |  |  |
| Intervention^1^ | -0.58 | -1.53, 0.37 | -0.77 | -1.72, 0.17 | -0.15 | -1.20, 0.91 |
| Total intrusions | 0.03 | 0.01, 0.05 | 0.03 | 0.05, 0.01 | 0.02 | 0.00, 0.03 |

^1^Reference is attention placebo control group.
